# Efficacy and mode of action of mesalazine in the treatment of diarrhoea-predominant irritable bowel syndrome (IBS-D): study protocol for a randomised controlled trial

**DOI:** 10.1186/1745-6215-14-10

**Published:** 2013-01-09

**Authors:** Matthew P Leighton, Ching Lam, Samir Mehta, Robin C Spiller

**Affiliations:** 1 Nottingham Clinical Trials Unit, University of Nottingham, Derby Road, Nottingham, NG7 2UH, UK; 2 Nottingham Digestive Diseases Centre-Biomedical Research Unit, University of Nottingham, Derby Road, Nottingham, NG7 2UH, UK

**Keywords:** Irritable bowel syndrome, Diarrhoea-predominant, Mesalazine

## Abstract

**Background:**

Irritable bowel syndrome (IBS) is reported by one in ten of the population accounting for up to 40% of new referrals to gastroenterology outpatients. Patients characteristically have abdominal discomfort and disturbed bowel habit. Diarrhoea-predominant IBS is characterised by frequent loose stools with associated urgency and abdominal cramps. Current symptomatic treatments can reduce bowel frequency but often fail to reduce discomfort.

Mesalazine is an anti-inflammatory drug used to treat patients with inflammatory bowel disease. There is one pilot study suggesting it may be beneficial to patients who have diarrhoea-predominant IBS but these findings need to be confirmed in a larger trial. The current study aims to test the effectiveness of mesalazine to reduce symptoms in diarrhoea-predominant IBS patients. The study will also investigate the mode of action of the drug, especially its impact on mast cell activation.

**Methods/design:**

This is a multicentre randomised, double-blind, placebo-controlled trial using a parallel group design. At least 108 participants with diarrhoea-predominant IBS will be recruited through at least six hospitals. The intervention is a 12-week course of 2g mesalazine granules taken up to twice a day. The comparator is a blinded placebo granule formulation.

Outcome measures include stool diaries, symptom questionnaires, stool and blood samples together with rectal mucosal biopsies. The daily stool diary will record stool frequency and form, urgency, bloating, abdominal pain and a global satisfaction with control of IBS scored each week. The questionnaires will assess bowel symptoms, while the samples and biopsies will be used to analyse underlying mechanisms of any response.

Primary outcome will be the average stool frequency during weeks 11 and 12 of the treatment period and will be compared between treatment arms using an analysis of covariance in the form of a general linear model incorporating baseline characteristics that are thought *a priori* to strongly predict outcome. The primary efficacy parameter will be the difference in mean frequency between treatment arms.

**Discussion:**

This report describes a randomised controlled trial that will provide evidence of any benefit of treating diarrhoea-predominant IBS patients with mesalazine. The results will be available toward the end of 2013.

**Trial registration:**

ISRCTN76612274

## Background

While the presenting complaints in IBS are usually abdominal pain and erratic bowel habits, at least half the IBS patients have an associated history of anxiety or depression and the presence of multiple unexplained physical symptoms otherwise known as ‘physical symptom disorder’ [1]. Patients often believe that stress aggravates their symptoms but there is a poor correlation between stress and symptoms on a day-to-day basis [2].

Evidence is accumulating that activation of mast cells can occur in stressed humans [3]. In diarrhoea-predominant irritable bowel syndrome (IBS-D) patients, mast cell numbers have been shown to be increased [4-7]. We propose that anxiety and chronic stressors increase the number of activated mast cells throughout the gut in IBS patients, thereby inducing the characteristic visceral hypersensitivity and abdominal pain. We hypothesise that mesalazine treatment, through its anti-inflammatory effects, will reduce the number of mast cells and, thereby, reduce abdominal pain and diarrhoea.

Older studies using cromoglycate, a mast cell stabiliser suggested benefit in those with evidence of allergy on skin prick testing but these studies were uncontrolled [8]. More recently there have been other smaller studies targeting mast cells with antihistamines such as ketotifen [9]. Although this reduced visceral hypersensitivity, it had no effect on mast cell numbers or release of mast cell mediators. Our own trial of prednisolone in post infective IBS showed no benefit for IBS symptoms but was of limited duration at just three weeks. It did however show a fall in mast cell numbers in patients on prednisolone compared to patients on placebo, but the difference was not significant probably because the study was underpowered [10]. A strategy that reduces mast cell numbers over the long term might well be more effective than specific inhibitors of mast cell activation or indeed any specific mast cell products since these are numerous, all with quite variable modes of action.

The first open-label trial of 12 patients with IBS-D who responded to mesalazine [7] showed a benefit that took about two to three months to become apparent. There have since been three further reports of open-label treatment [11,12] and two small randomised control trials [13,14]. All except the Corinaldesi trial [13] used patients with IBS-D. The Bafutto trial showed a reduction in stool frequency, stool consistency and abdominal pain but was uncontrolled [12]. The Andrews study involved just six patients but this showed that mesalazine decreased biopsy proteolytic activity. The Corinaldesi trial in unselected IBS patients showed a significant reduction of mast cell numbers, an overall reduction in inflammatory cells [13], and an improvement in general well being without altering bowel function significantly.

## Rationale for the current study

Studies in Nottingham over the last decade demonstrated the importance of anxiety and depression [15], which along with adverse life events, increase the risk of post-infective IBS (PI-IBS) [16]. The changes observed in PI-IBS are very similar to those in IBS-D, the predominant bowel disturbance being diarrhoea with a similar prognosis [17]. This work has been supported by others who have shown inflammatory changes in IBS-D patients who do not have a background of previous infection [18,19]. Increased gut permeability has also been shown in IBS-D [20], a feature thought to be the result of psychological stress and/or inflammation secondary to infection [21].

We hypothesise that, through its anti-inflammatory effects, mesalazine treatment will reduce abdominal pain and diarrhoea by reducing the number of mast cells and subsequent release of their mediators which increase gut permeability and sensitivity [22]. Mesalazine therefore, both by virtue of inhibiting other inflammatory pathways and by directly inhibiting mast cell pathways, may reduce mucosal immune activation. We plan to investigate the effect of long-term mesalazine on mast cell numbers, the chronic inflammatory cells, and the mucosal production of inflammatory cytokines IL-1β and TNF-α, as well as the mast cell enzyme tryptase.

## Methods/design

### Trial purpose

The purpose of the trial is to define the clinical benefit and possible mediators of the benefit of mesalazine in IBS with diarrhoea.

We will therefore evaluate symptoms (primarily bowel frequency) and markers reflecting mast cell activation and small bowel tone.

### Trial design

**Figure 1:**
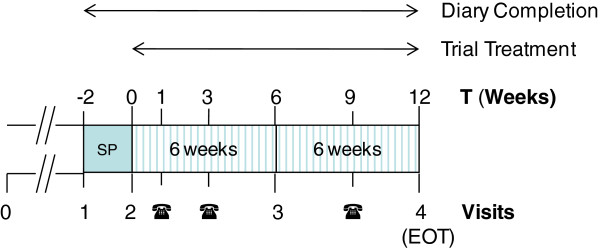
Participant involvement.

This is a multicentre, two-arm, parallel group, double-blind, randomised placebo- controlled trial comparing mesalazine with placebo in patients with diarrhoea-predominant irritable bowel syndrome. Trial consists of a screening visit to check initial eligibility and to take consent prior to the participant completing a two-week screening phase stool diary. If they continue to remain eligible they will be randomised to receive 12 weeks of active or placebo treatment, stratified by site. Both groups continue completing the stool diary during treatment and will have three telephone contacts and two visits prior to completion. Participants will be in the study for 14 weeks. Please see Figure 1 for more details.

### Trial objectives

The trial objectives were as follows:

1) To assess the effect of mesalazine on stool frequency.

2) To assess the effect of mesalazine on

·• Overall IBS symptoms

·• Mast cell numbers, mucosal lymphocytes and faecal tryptase activity

·• Release of mast cell mediators from rectal biopsies.

·• Small bowel tone by measurement of fasting small bowel water content using magnetic resonance imaging (MRI).

·•To assess ability of biomarkers (mucosal/MRI parameters) to predict treatment response

### Participant recruitment

Ethical approval for the study was obtained from the Oxfordshire Research Ethics Committee B, United Kingdom.

Participants were recruited from IBS clinics at the investigator’s hospital, or from lists of patients who have previously taken part in research studies and have indicated that they would like to be contacted about future relevant research projects. In addition, in conjunction with the local Primary Care Research Network, we approached general practitioners (GPs) to ask them to search their databases for eligible participants and send out letters of invitation along with a participant information sheet (PIS). Either way, the initial approach was from a member of the patient’s usual care team or from appropriately authorised research nurses. We also advertised in the local newspaper and information about the study was also displayed in the relevant clinical areas. Ethical approval was obtained for all advertisements or posters displayed.

Patients were eligible for the study if they provided written informed consent and if they: 1) were male or female patients aged 18 to 75 years; 2) had had a colonoscopy or sigmoidoscopy within the last 12 months to exclude microscopic or any inflammatory colitis. (If not, but they have had a negative colonoscopy within five years and symptoms are unchanged, then a sigmoidoscopy and mucosal biopsy of the left colon was deemed to be sufficient to exclude microscopic or any inflammatory colitis); 3) met Rome III criteria for IBS-D prior to screening phase; 4) had ≥25% soft (Bristol Stool Form Score [BSFS] score >4) and <25% hard (BSFS 1 or 2) stools during the screening phase, as indicated in the daily symptom and stool diary; 5) had a stool frequency of three or more per day for two or more days per week during the screening phase; 6) had satisfactorily completed the daily stool and symptom diary during the screening phase at the discretion of the investigator; and 7) if female and of child-bearing potential, willing and able to use at least one highly effective contraceptive method throughout the study. If (4) and/or (5) is/are not met but the results are considered atypical (as observed from medical history and patient recall) then the patient can be rescreen on one occasion only.

Rome III criteria for IBS-D [23] were used to evaluate effect of mesalazine on patients during course of study. These criteria are as follows:

Abdominal Pain or discomfort at least two to three days/month in the last three months (criterion fulfilled for the last three months with symptom onset at least six months prior to screening) associated with two or more of the following:

1) Improvement with defecation;

2) Onset associated with a change of stool frequency;

3) Onset associated with a change in form (appearance) of stool.

Patients were not eligible for the study if they: 1) were pregnant or breast feeding; 2) had prior abdominal surgery which may cause bowel symptoms similar to IBS (note appendectomy and cholecystectomy will not be an exclusion); 3) were unable to stop anti-muscarinics, anti-spasmodics, high-dose tricyclic antidepressants (that is, above 50 mg/day), opiates/anti-diarrhoeal drugs, NS-AIDs (occasional over the counter use and topical formulations were allowed), long-term antibiotics, other anti-inflammatory drugs or 5-ASA containing drugs; 4) were on selective serotonin re-uptake inhibitors and low-dose tricyclic antidepressants (that is, up to 50 mg/day) for at least three months previous and unwilling to remain on a stable dose for the duration of the trial; 5) had other gastro-intestinal diseases including colitis and Crohn’s disease; 6) had renal impairment, severe hepatic impairment or salicylate hypersensitivity; 7) were currently participating in another trial or had been in a trial within the previous three months; 8) in the opinion of the investigator were considered unsuitable due to inability to comply with instructions or 9) had serious concomitant diseases (for example, cardiovascular, respi-ratory, neurological *et cetera*).

Regarding (3) loperamide was allowed as a rescue medication throughout the trial, however if more than two doses/week were taken during the screening phase then the patients became ineligible, though they could be re-screened on one occasion only.

### The interventions

#### Mesalazine and placebo

A licensed mesalazine slow-release granule formulation (2g) was used (PENTASA®, Ferring Pharmaceuticals Ltd, West Drayton, United Kingdom.) A matching placebo granule formulation was also used. In order to maintain blinding, active and placebo granules were packaged in matching de-identified trial-specific foil sachets.

The trial dose was 2g once a day (one dose in the morning) for the first week of treatment, then, if the initial dose was tolerated, a step increase to 2g twice a day (one dose in the morning, one dose in the evening) for the remaining 11 weeks of treatment. If the current dose was not tolerated participants could reduce that dose or stop completely.

All dose adjustments were made in consultation with the local investigator. Compliance was recorded in the daily stool diary and from returned sachets.

### Randomisation and blinding

This was a double-blind study. Neither participant nor supervising doctor nor study nurse was aware of the treatment allocation.

Randomisation was based on a computer-generated pseudo-random code using random permuted bal-anced blocks of randomly varying size, created by the Nottingham Clinical Trials Unit (NCTU). The randomization was stratified by site with participants randomly allocated (1:1 ratio) to either mesalazine or placebo. Access to the master sequence of treatment allocations was confined to the NCTU IT Manager and the Nottingham University Hospitals NHS Trust Clinical Trials pharmacy.

Investigators/sites accessed the treatment allocation for each participant by means of a remote, secure, internet-based randomisation system developed and maintained by the NCTU. The sequence of treatment allocations was concealed until interventions had all been assigned and recruitment, data collection, and all other trial-related assessments were complete.

In the event of the need to break the code, usually due to clinical need, the date and reason for breaking the code were recorded in the eCRF. The code could only be broken by authorised personnel via the web-based eCRF or, in the event the system being inaccessible, the Nottingham University Hospitals NHS Trust Clinical Trials pharmacy kept a copy of the treatment allocations.

### Primary endpoint

#### Clinical endpoint

1) Difference in average stool frequency during weeks 11–12 of the treatment period.

#### Mechanistic endpoint

1) Difference in mast cell numbers per mm^2^ during weeks 11–12 of the treatment period.

#### Secondary endpoint

Clinical secondary endpoints during weeks 11 and 12 of the treatment period:

1) Average daily severity of abdominal pain on a 0 to 10 scale.

2) Days with urgency.

3) Mean stool consistency using Bristol Stool Form Score.

4) Global satisfaction with control of IBS symptoms as assessed from the answer to the question ‘Have you had satisfactory relief of your IBS symptoms this week? Yes/No.’

Mechanistic secondary endpoints during weeks 11 and 12 of the treatment period:

1) Mast cell tryptase release during 6-hour biopsy incubation

2) IL-1β, TNF-α, histamine and serotonin secretion during same incubation

3) Small bowel tone assessed by volume of fasting small bowel water

4) Fecal tryptase activity

5) Difference in primary outcome measure between those with different TNFSF15 polymorphism

Ancillary secondary endpoints during weeks 11 and 12 of the treatment period:

1) EuroQol-5D (EQ-5D)

2) Centers for Disease Control and Prevention Health-Related Quality of Life (CDC HRQOL4)

3) Hospital, Anxiety and Depression Scale (HADS)

4) Patient Health Questionnaire-15 (PHQ-15)

### Outcome measures

#### Daily symptom and stool diary

Participants completed a daily symptom and stool diary throughout their participation indicating hours of pain, severity of abdominal pain on a 0 to 10 scale, presence of urgency (yes/no) and bloating on a 0–10 scale. They were also asked on a weekly basis the following yes/no question: ‘Have you had satisfactory relief of your IBS symptoms this week?’

#### IBS-D sub-groups

The Rome III criteria questionnaire will be used to assess whether the participant has IBS-D. In addition the participants will answer supplementary questions to identify any cases that are post-infectious IBS-D.

#### Psychological profiles

We used the HADS and PHQ-15 questionnaires, collected at baseline and 12 weeks.

#### Quality of life questionnaires

Quality of Life was assessed using the two generic quality of life measures that had been validated in IBS patient groups: the CDC HRQOL4 [24] and the EQ-5D [25], which has been shown to be sensitive to a successful treatment of symptoms [26].

#### MRI (Nottingham only)

Fasting small bowel water content was assessed using our previously validated MRI technique [27]. The volume of small bowel water gives an indirect assessment of intestinal tone. We have previously shown that small bowel water content is significantly reduced in IBS-D, both fasting and postprandial [28].

#### Flexible Sigmoidoscopy and biopsies (Nottingham only

Patients will undergo an unsedated flexible sigmoidoscopy with biopsy of the sigmoid colon. Biopsies will be taken to investigate mast cells, mRNA for IL-1, TNF-α and protein levels, as well as for tryptase, serotonin and histamine release on incubation of mucosal biopsies.

#### Stool sample

Stool samples will be analysed for proteases and other inflammation biomarkers. Bacterial DNA isolated from stool samples will also be analysed using the HITChip [29].

#### Blood sample

Serum isolated from blood samples were analysed for inflammation biomarkers. DNA isolated from blood samples were analysed for any genetic link between polymorphisms and treatment response.

### Sample size

Our previous study on diarrhoea-predominant IBS patients gives a mean stool frequency of 3.1 (standard deviation 2.0). Tuteja and colleagues reported mesalazine decreasing stool frequency by 1.4 bowel movements per day [14]. We calculated from these data that 108 completed patients (randomized 1:1 to mesalazine or placebo) would give the study an 80% power to detect such an effect at the 1% significance level (90% at 5% significance). As we had not accounted for attrition if participants withdrew, these participants were replaced in order to ensure 108 completed participants.

Much smaller numbers would be needed to assess the effect of mesalazine on mast cell numbers and tryptase release. Corinaldesi *et al*. reported a 36% decrease in mast cell numbers from mean 9.2 (standard deviation 2.5) [13], which would have required just 12 patients to show such a decrease with a power of 90% at the 1% significance level.

### Statistical analysis

The primary analysis will be performed on the full analysis set (that is, all randomised participants for whom the primary endpoint (symptoms at 11 and 12 weeks) is available) using an analysis of covariance in the form of a general linear model incorporating terms for baseline frequency, treatment arm, centre, and other baseline characteristics which are thought a priori to strongly predict outcome.

Secondary and mechanistic endpoints were treated similarly, after transformation to approximate normality as required for continuous variables, while binary and count outcomes were handled by logistic or Poisson regression, as appropriate. All analyses were performed using the current version of STATA (StataCorp, Texas, USA), adopting the intention to treat principle following multiple imputation for missing data (with a sensitivity analysis for the missing at random assumption for the primary outcome). For mechanistic variables a per-protocol analysis was additionally performed.

No interim or subgroup analyses were planned for efficacy.

Assessment of safety followed the same principles as the assessment of efficacy except that the safety set (that is, all randomised participants who receive at least one dose of the study drug) were used.

Procedures for missing, unused and spurious data were handled by multiple imputation using the method of chained equations.

### Assessment of adverse events

This trial is using a drug, mesalazine, for which the side-effect profile is well established. Therefore the study specific definition of an adverse event was:

1) any study drug-related event as listed as a known side effects of mesalazine with the exception of diarrhoea and abdominal pain, which will only be recorded if these disease symptoms are exacerbated.

OR

2) any condition detected or diagnosed after the medicinal product has been administered and has a possible, probable or definite causal relationship with the study drug.

Due to the study specific definition the recording of adverse events and serious adverse event reporting began following the 1st dose of study treatment.

### Definition of a protocol deviation

The following were predefined protocol violations with a direct bearing on the primary endpoint:

1) Taking of rescue medication (loperamide) during the primary endpoint assessment period (that is, weeks 11 and 12 of the treatment period).

2) Taking of antibiotics during the primary endpoint assessment period (that is, weeks 11and 12 of the treatment period).

This summary paper was based on protocol version 3.0, 5th May 2011. A copy of the full protocol is available on request.

## Trial status

Recruitment started in March 2011 and is anticipated to continue until May 2013.

## Abbreviations

BSFS: Bristol stool and form score; CDC-HRQOL: Centers for Disease Control and Prevention Health-Related Quality of Life; eCRF: Electronic case report form; EQ-5D: EuroQol-5D; GPs: General practitioners; HADS: Hospital anxiety and depression scale; IBS: Irritable bowel syndrome; IBS-D: Irritable bowel syndrome - diarrhea predominant; MRI: Magnetic resonance imaging; NCTU: Nottingham Clinical Trials Unit; NDDC-BRU: Nottingham Digestive Diseases Centre-Biomedical Research Unit; PHQ-15: Patient health questionnaire-15; PIS: Participant information sheet; PI-IBS: Post-infective irritable bowel syndrome.

## Competing interests

None of the authors have any competing interests.

## Authors’ contributions

RS conceived the study, was the grant holder and chief investigator for the study, wrote this summary and wrote the original protocol. ML, based in the Nottingham Clinical Trials unit (NCTU), and CL, based in the NDDC-BRU, were responsible for day-to-day running of the trial and data collection/management and have been responsible for most revisions of the protocol incorporating suggestions and comments from RS. In addition SM is the study statistician and has responsibility for the analysis. All authors read and approved the final manuscript.

## References

[B1] Kroenke K Physical symptom disorder: a simpler diagnostic category for somatization-spectrum conditions J Psychosom Res 2006 60 335 339 10.1016/j.jpsychores.2006.01.022 16581354

[B2] Whitehead WE Crowell MD Robinson JC Heller BR Schuster MM Effects of stressful life events on bowel symptoms: subjects with irritable bowel syndrome compared with subjects without bowel dysfunction Gut 1992 33 825 830 10.1136/gut.33.6.825 1624167 PMC1379344

[B3] Santos J Saperas E Nogueiras C Mourelle M Antolin M Cadahia A Malagelada JR Release of mast cell mediators into the jejunum by cold pain stress in humans Gastroenterology 1998 114 640 648 10.1016/S0016-5085(98)70577-3 9516384

[B4] Guilarte M Santos J de Torres I Alonso C Vicario M Ramos L Martínez C Casellas F Saperas E Malagelada JR Diarrhoea-predominant IBS patients show mast cell activation and hyperplasia in the jejunum Gut 2007 56 203 209 10.1136/gut.2006.100594 17005763 PMC1856785

[B5] Park JH Rhee PL Kim HS Lee JH Kim YH Kim JJ Rhee JC Mucosal mast cell counts correlate with visceral hypersensitivity in patients with diarrhea predominant irritable bowel syndrome J Gastroenterol Hepatol 2006 21 71 78 10.1111/j.1440-1746.2005.04143.x 16706815

[B6] Dunlop SP Jenkins D Spiller RC Distinctive clinical, psychological, and histological features of postinfective irritable bowel syndrome Am J Gastroenterol 2003 98 1578 1583 10.1111/j.1572-0241.2003.07542.x 12873581

[B7] Aron J Response to mesalamine and balsalazide in patients with irritable bowel syndrome refractory to alosetron and tegaserod [abstract] Gastroenterology 2005 128 A329 A330

[B8] Stefanini GF Prati E Albini MC Piccinini G Capelli S Castelli E Mazzetti M Gasbarrini G Oral disodium cromoglycate treatment on irritable bowel syndrome: an open study on 101 subjects with diarrheic type Am J Gastroenterol 1992 87 55 57 1728124

[B9] Klooker TK Braak B Koopman KE Welting O Wouters MM van der Heide S Schemann M Bischoff SC van den Wijngaard RM Boeckxstaens GE The mast cell stabiliser ketotifen decreases visceral hypersensitivity and improves intestinal symptoms in patients with irritable bowel syndrome Gut 2010 59 1213 1221 10.1136/gut.2010.213108 20650926

[B10] Dunlop SP Jenkins D Neal KR Naesdal J Borgaonker M Collins SM Spiller RC Randomized, double-blind, placebo-controlled trial of prednisolone in post-infectious irritable bowel syndrome Aliment Pharmacol Ther 2003 18 77 84 10.1046/j.1365-2036.2003.01640.x 12848628

[B11] Andrews CN Petcu R Griffiths T Bjarnason J Chapman K Cellars L Vergnolle N Roux KP Mesalamine alters colonic mucosal proteolytic activity and fecal bacterial profiles in diarrhea-predominant irritable bowel syndrome (IBS-D) Gastroenterology 2008 134 A548-A

[B12] Bafutto M Almeida JR Almeida RC Almeida TC Leite NV Filho JR Treatment of post-infectious irritable bowel syndrome and non infective irritable bowel syndrome with mesalazine [abstract] Gastroenterology 2008 134 A672

[B13] Corinaldesi R Stanghellini V Cremon C Gargano L Cogliandro RF De Giorgio R Bartesaghi G Canovi B Barbara G Effect of mesalazine on mucosal immune biomarkers in irritable bowel syndrome: a randomized controlled proof-of-concept study Aliment Pharmacol Ther 2009 30 245 252 10.1111/j.1365-2036.2009.04041.x 19438846

[B14] Tuteja A Hale D Stoddard G Fang J Tolman K Double-blind placebo controlled study of mesalamine in post-infective irritable bowel syndrome [abstract] Am J Gastroenterol 2008 103 S480

[B15] Dunlop SP Jenkins D Neal KR Spiller RC Relative importance of enterochromaffin cell hyperplasia, anxiety, and depression in postinfectious IBS Gastroenterology 2003 125 1651 1659 10.1053/j.gastro.2003.09.028 14724817

[B16] Gwee KA Leong YL Graham C McKendrick MW Collins SM Walters SJ Underwood JE Read NW The role of psychological and biological factors in postinfective gut dysfunction Gut 1999 44 400 406 10.1136/gut.44.3.400 10026328 PMC1727402

[B17] Neal KR Barker L Spiller RC Prognosis in post-infective irritable bowel syndrome: a six year follow up study Gut 2002 51 410 413 10.1136/gut.51.3.410 12171965 PMC1773359

[B18] Chadwick VS Chen W Shu D Paulus B Bethwaite P Tie A Wilson I Activation of the mucosal immune system in irritable bowel syndrome Gastroenterology 2002 122 1778 1783 10.1053/gast.2002.33579 12055584

[B19] Liebregts T Adam B Bredack C Roth A Heinzel S Lester S Downie-Doyle S Smith E Drew P Talley NJ Holtmann G Immune activation in patients with irritable bowel syndrome Gastroenterology 2007 132 913 920 10.1053/j.gastro.2007.01.046 17383420

[B20] Dunlop SP Hebden J Campbell E Naesdal J Olbe L Perkins AC Spiller RC Abnormal intestinal permeability in subgroups of diarrhea-predominant irritable bowel syndromes Am J Gastroenterol 2006 101 1288 1294 10.1111/j.1572-0241.2006.00672.x 16771951

[B21] Spiller R Garsed K Postinfectious irritable bowel syndrome Gastroenterology 2009 136 1979 1988 10.1053/j.gastro.2009.02.074 19457422

[B22] Fox CC Moore WC Lichtenstein LM Modulation of mediator release from human intestinal mast cells by sulfasalazine and 5-aminosalicylic acid Dig Dis Sci 1991 36 179 184 10.1007/BF01300753 1703070

[B23] Longstreth GF Thompson WG Chey WD Houghton LA Mearin F Spiller RC Functional bowel disorders Gastroenterology 2006 130 1480 1491 10.1053/j.gastro.2005.11.061 16678561

[B24] Lackner JM Gudleski GD Zack MM Katz LA Powell C Krasner S Holmes E Dorscheimer K Measuring health-related quality of life in patients with irritable bowel syndrome: can less be more? Psychosom Med 2006 68 312 320 10.1097/01.psy.0000204897.25745.7c 16554399

[B25] Groll D Vanner SJ Depew WT DaCosta LR Simon JB Groll A Roblin N Paterson WG The IBS-36: a new quality of life measure for irritable bowel syndrome Am J Gastroenterol 2002 97 962 971 10.1111/j.1572-0241.2002.05616.x 12003433

[B26] Bracco A Jonsson B Ricci JF Drummond M Nyhlin H Economic evaluation of tegaserod vs. placebo in the treatment of patients with irritable bowel syndrome: an analysis of the TENOR study Value Health 2007 10 238 246 10.1111/j.1524-4733.2007.00179.x 17645678

[B27] Hoad CL Marciani L Foley S Totman JJ Wright J Bush D Cox EF Campbell E Spiller RC Gowland PA Non-invasive quantification of small bowel water content by MRI: a validation study Phys Med Biol 2007 52 6909 6922 10.1088/0031-9155/52/23/009 18029983

[B28] Marciani L Cox EF Hoad CL Pritchard S Totman JJ Foley S Mistry A Evans S Gowland PA Spiller RC Postprandial changes in small bowel water content in healthy subjects and patients with irritable bowel syndrome Gastroenterology 2010 138 469 477 10.1053/j.gastro.2009.10.055 19909743

[B29] Arumugam M Raes J Pelletier E Le Paslier D Yamada T Mende DR Fernandes GR Tap J Bruls T Batto JM Bertalan M Borruel N Casellas F Fernandez L Gautier L Hansen T Hattori M Hayashi T Kleerebezem M Kurokawa K Leclerc M Levenez F Manichanh C Nielsen HB Nielsen T Pons N Poulain J Qin J Sicheritz-Ponten T Tims S Enterotypes of the human gut microbiome Nature 2011 473 174 180 10.1038/nature09944 21508958 PMC3728647

